# The Complete Mitochondrial Genome of *Glyptothorax macromaculatus* Provides a Well-Resolved Molecular Phylogeny of the Chinese Sisorid Catfishes

**DOI:** 10.3390/genes9060282

**Published:** 2018-06-04

**Authors:** Yunyun Lv, Yanping Li, Zhiqiang Ruan, Chao Bian, Xinxin You, Junxing Yang, Wansheng Jiang, Qiong Shi

**Affiliations:** 1 BGI Education Center, University of Chinese Academy of Sciences, Shenzhen 518083, China; lvyunyun@genomics.cn (Y.L.); youxinxin@genomics.cn (X.Y.); 2 Shenzhen Key Lab of Marine Genomics, Guangdong Provincial Key Lab of Molecular Breeding in Marine Economic Animals, BGI Academy of Marine Sciences, BGI Marine, BGI, Shenzhen 518083, China; liyanping@genomics.cn (Y.L.); ruanzhiqiang@genomics.cn (Z.R.); bianchao@genomics.cn (C.B.); 3 State Key Laboratory of Genetic Resources and Evolution, Kunming Institute of Zoology, Chinese Academy of Sciences, Kunming 650223, China; yangjx@mail.kiz.ac.cn (J.Y.); jiangws@mail.kiz.ac.cn (W.J.); 4 Laboratory of Aquatic Genomics, College of Life Sciences and Oceanography, Shenzhen University, Shenzhen 518060, China

**Keywords:** Sisoridae, mitochondrial genome, evolution, selection, high-elevation adaption

## Abstract

Previous phylogenetic analyses of the Chinese sisorid catfishes have either been poorly resolved or have not included all the 12 sisorid genera. Here, we successfully assembled the first complete mitochondrial genome of the sisorid fish *Glyptothorax macromaculatus*. Based on this novel mitochondrial genome and previously published mitochondrial genomes in the Sisoridae, we generated maximum likelihood and Bayesian phylogenies. We dated our preferred topology using fossil calibration points. We also tested the protein-coding genes in the mitochondrial genomes of the glyptosternoid fishes for signals of natural selection by comparing the nucleotide substitution rate along the branch ancestral to the glyptosternoid fishes to other branches in our topology. The mitochondrial sequence structure of *G. macromaculatus* was similar to those known from other vertebrates, with some slight differences. Our sisorid phylogenies were well-resolved and well-supported, with exact congruence between the different phylogenetic methods. This robust phylogeny clarified the relationships among the Chinese sisorid genera and strongly supported the division of the family into three main clades. Interestingly, the glyptosternoid divergence time predicted by our molecular dating analysis coincided with the uplift of the Tibetan Plateau, suggesting that geology may have influenced speciation in the Sisoridae. Among the mitochondrial protein-coding genes, *atp8* may have most rapidly evolved, and *atp6* may have been subjected to positive selection pressure to adapt to high elevations. In summary, this study provided novel insights into the phylogeny, evolution and high-altitude adaptions of the Chinese sisorid fishes.

## 1. Introduction

The sisorid catfishes (family Sisoridae) are widely distributed in the Southwest of China. The Sisoridae are classified into 12 genera, including *Bagarius*, *Creteuchiloglanis*, *Euchiloglanis*, *Exostoma*, *Gagata*, *Glaridoglanis*, *Glyptosternon*, *Glyptothorax*, *Oreoglanis*, *Pareuchiloglanis*, *Pseudecheneis*, and *Pseudexostoma* [1]. With the exception of four genera (*Bagarius*, *Gagata*, *Glyptothorax*, and *Pseudecheneis*), the sisorids are glyptosternoid fishes that are unevenly distributed across the Tibetan Plateau [2]. The glyptosternoid fishes are thus obligate inhabitants of high altitudes.

Morphology-based classifications of the sisorid fishes may be inaccurate as these species are morphologically similar [3], although previous molecular phylogenies have recovered well-supported species-level relationships in this family, as well as the monophyly of Sisoridae [3] and glyptosternoid fishes [4]. The monophyly of Sisoridae was supported by an analysis of 16S rRNA [3], and the glyptosternoid fishes were supported as a monophyletic group based on cytochrome b (*cytb*) and 16S rRNA [4]. The genera *Glyptosternon* and *Exostoma* were recovered as basal to the other glyptosternoid genera based on an analysis of *cytb* [5]. These molecular studies have contributed greatly to a better understanding of the phylogenetic relationships among the sisorid fishes.

Unfortunately, aspects of several of these previously published phylogenies were incongruent. For example, the genus *Pseudecheneis* clustered with the glyptosternoids within Guo et al., (2004, 2007) [3,6], but fell sister to the glyptosternoids in Guo et al., (2005) [4]. *Pseudecheneis* was recovered basal to all other sisorid genera by both Peng et al., (2004, 2006) and Yu & He (2012) [5,7,8]. *Pareuchiloglanis* was paraphyletic in Peng et al., (2006) [8], but not in Yu & He (2012) [7]. These conflicting phylogenies were weakly supported, possibly resulting from the use of inadequate genetic information. Recently, a well-supported molecular phylogeny of the Sisoridae, based on the complete mitochondrial genomes of a few sisorid species, has become available [9,10]. However, a robust phylogeny including all 12 genera of the Chinese sisorid catfishes remains unavailable. This knowledge gap prevents a deeper understanding of phylogenetic relationships among the sisorid fishes.

In addition to the determination of phylogenetic relationships, it is also interesting to study the evolution of protein-coding mitochondrial genes in the obligate high-elevation glyptosternoid fishes. Based on the rates of synonymous (*Ks*) and nonsynonymous (*Ka*) substitutions in mitochondrial protein-coding genes, it was shown that mitochondrial encoded cytochrome c oxidase 1 (*cox1*) has been subjected to positive selection in the glyptosternoid fishes; this enzyme encoded by this gene is part of the electron transport chain for mitochondrial oxidative phosphorylation [10]. Therefore, it is possible that, in these high-altitude fishes, some mitochondrial protein-coding genes may be under selection pressure to increase energy consumption. However, other factors may also significantly increase the *Ka/Ks* ratio, such as effective population size [11] and life history [12,13].

In this study, we focused on the sisorid fish *Glyptothorax macromaculatus*. We aimed to assemble and delineate a complete mitochondrial genome for this species and to investigate the arrangement of genes within this genome. We then aimed to integrate this genome with previously published mitochondrial genomes representing the 12 genera of Chinese sisorid catfishes and to use this relatively large dataset to investigate the phylogenetic relationships and species divergence times in the Sisoridae. Finally, we aimed to determine the substitution rates of protein-coding genes based on the complete mitochondrial genome and to identify signals of positive selection.

## 2. Materials and Methods

### 2.1. Sampling and Complete Mitochondrial Genome Assembly of G. macromaculatus

We collected an adult specimen of *G. macromaculatus* in Yunnan Province, China. This voucher specimen was deposited in the Marine Bank of the China National GeneBank (Voucher no. YN170702001_FI), Shenzhen, China. We extracted total DNA from the fin muscle using a Puregene Tissue Core Kit A (Qiagen, Germantown, MD, USA). With traditional whole-genome shotgun sequencing, we constructed a series of short-insert libraries (270, 500, and 800 bp) and long-insert libraries (2, 5, 10, and 20 kb) using standard protocols (Illumina, San Diego, CA, USA). Paired-end sequencing was performed on an Illumina Hiseq2500 platform (San Diego, CA, USA) at BGI (Shenzhen, China). To construct the sequence library, we assembled a roughly complete mitochondrial genome of *G. macromaculatus*. In brief, we used the mitochondrial genome of a congeneric, *Glyptothorax trilineatus* (downloaded from National Center for Biotechnology Information (NCBI) with GenBank accession no. NC_021608.1; Table S1). We then identified reads highly similar to the reference mitochondrial genome with SOAP2 (version 2.21, [14]). All highly similar reads were assembled with SPAdes (version 3.10.0, [15]). Finally, the assembled result was compared to the reference genome with Blast (version 2.6.1, [16,17]), and incomplete regions were filled with sequences generated by subsequent molecular amplification as described below.

We amplified incomplete regions using polymerase chain reactions (PCRs). We designed 12 related primer pairs (Table S2) with Primer Premier 5 (Premier Biosoft, Palo Alto, CA, USA) PCRs were performed in 50 μL volumes, each containing 5 μL of 10× buffer (with Mg^2+^), 4 μL of 2.5 mM dNTPs, 1 U of Taq DNA polymerase (rTaq; TaKaRa, Dalian, China), 1 μL of each primer (10 μM), and 1 μL of genomic DNA (100 ng/μL), made up to 50 μL with double-distilled water. We used the following PCR cycling conditions: pre-denaturation at 94 °C for 5 min; 35 cycles of denaturation at 94 °C for 30 s, annealing at 55 °C for 30 s, and elongation at 72 °C for 2 min, and a final elongation at 72 °C for 10 min. All PCRs were conducted in a Veriti Thermal Cycler (Applied Biosystems, Carlsbad, CA, USA). PCR products were checked via electrophoresis on 1% agarose gels and were subsequently purified using Qiagen Gel Extraction Kits (Qiagen China, Shanghai, China). Both strands of each product were sequenced using the previously designed PCR primer pairs (Table S2).

After all incomplete regions had been successfully amplified, we combined these sequences with the previously assembled incomplete mitochondrial genome to generate the complete mitochondrial genome of *G. macromaculatus*. We subsequently annotated coding genes, RNA, and D-loop regions based on BLAST comparisons with the reference genome.

### 2.2. Phylogenetic Analyses and Divergence Time Estimation

The complete mitochondrial genomes of 23 sisorid fishes were downloaded from NCBI GenBank (Table S1). Including the novel *G. macromaculatus* genome, our analysis included all 12 currently described genera of Chinese sisorid catfishes. We used three species of the Amblycipitidae (in the same superfamily as the Sisoridae) as outgroups [5,8,9,10]. Therefore, the complete dataset used for phylogenetic analysis included 27 complete mitochondrial genomes (Table S1).

All sequences were aligned with MAFFT (version 5) [18]. As some sites were difficult to align with confidence, we eliminated those poorly aligned positions with Gblocks (version 0.91b), using default parameters [19]. We estimated the best nucleotide substitution model for our alignment with jModelTest (version 2.1.5, [20]), using the AIC (Akaike Information Criterion). We used the best nucleotide substitution model (GTR + G + I) for maximum likelihood (ML) analysis in PhyML (version 3.1) [21] and RAxML (version 8.0.17) [22], with 1000 replicates to estimate node support. To confirm the topology generated by ML, we also analyzed our alignment using Bayesian inference (BI) in MrBayes (version 3.2.2, [23]), with the same nucleotide substitution model (GTR + G + I). We performed two parallel runs for 2 M generations (four chains per run), sampling every 500 generations. The initial 25% of the runs were discarded as burn-in. We identified the maximum clade credibility tree from the remaining topologies using TreeAnnotator (version 1.7.5) [24].

After phylogenetic tree construction, we estimated the molecular timing of species divergences with the Bayesian method MCMCTree in the PAML package (version 4.9e) [25]. Three nodes (C1, C2, and C3) were considered time-calibrated points with normal distributions and soft constraint bands (allowing a small probability (0.025) of violation). Following a previous analysis [8], the C1 calibration point was estimated based on a fossil of *Bagarius yarrelli* dating from the Pliocene (~5.3–1.8 million years ago (Ma)). The C2 calibration point was estimated to be the most recent common ancestor (MRCA) of the Sisoridae (~12.9–8 Ma), while the C3 calibration point was estimated to be the MRCA of the glyptosternoids (~9.8–6.1 Ma). We used 100,000 samples for the Markov chain Monte Carlo (MCMC) analysis, discarding the first 20% of all samples as burn-in. An independent rates model (clock = 2), which follows a lognormal distribution, was used for the MCMC search.

### 2.3. Substitution Rate Estimation and Comparison

*Ka*/*Ks* ratios may differ over evolutionary time among mitochondrial coding genes. To understand the average changes in nucleotide substitution rates in the glyptosternoids, we calculated the average *Ka* and *Ka*/*Ks* values across all 13 mitochondrial protein-coding genes (*atp6*, *atp8*, *cox1*, *cox2*, *cox3*, *cytb*, *nd1*, *nd2*, *nd3*, *nd4*, *nd4l*, *nd5* and *nd6*). Based on the annotations of the 24 sisorid mitochondrial genomes, we extracted each of the 13 genes, and aligned each gene separately using the codon-based model in the Muscle module of MEGA (version 7) [26]. We calculated pairwise *Ka* and *Ka*/*Ks* values between each pair of single-gene datasets in DnaSP (version 5.0) [27]. The average values of *Ka* and *Ka*/*Ks* were calculated in R [28], and were used to represent changes of each coding gene’s substitution rates.

As glyptosternoid fishes are mainly distributed in the Hengduan Mountains [5,7,8,9,10], we focused on changes of the nucleotide substitution rate in the MRCA of this group. To test if positive selection may have occurred in the MRCA of this obligate high-altitude group, we compared the *Ka*/*Ks* values along the ancestral glyptosternoid branch to other branches. The topology of evolutionary tree used in this analysis was derived from the species-level phylogeny constructed above. We first ran a one-ratio model (model = 0; assuming a unique ω0 (*Ka*/*Ks*) ratio along all branches of the phylogenetic tree) for each of the 13 protein-coding mitochondrial genes with the CODEML modules of PAML. We next ran a two-ratio model for each of the 13 protein-coding genes (model = 2; setting the glyptosternoids branch as the independently-changing target, ω2; setting all other branches as the constant ω1). Likelihood ratio (LR) and Chi-square (χ^2^) tests comparing the probabilities of the one-ratio and two-ratio models were performed for all 13 genes. If the two-ratio model had a significantly higher probability than the one-ratio model for a given gene, we applied a branch-site model to that gene (assuming each sequence position might have an independent ω) to test which sites had been subject to positive selection. Based on these results, we identified signals of positive selection on the ancestral glyptosternoid branch.

## 3. Results

### 3.1. The Complete Mitochondrial Genome of G. macromaculatus

**Figure 1:**
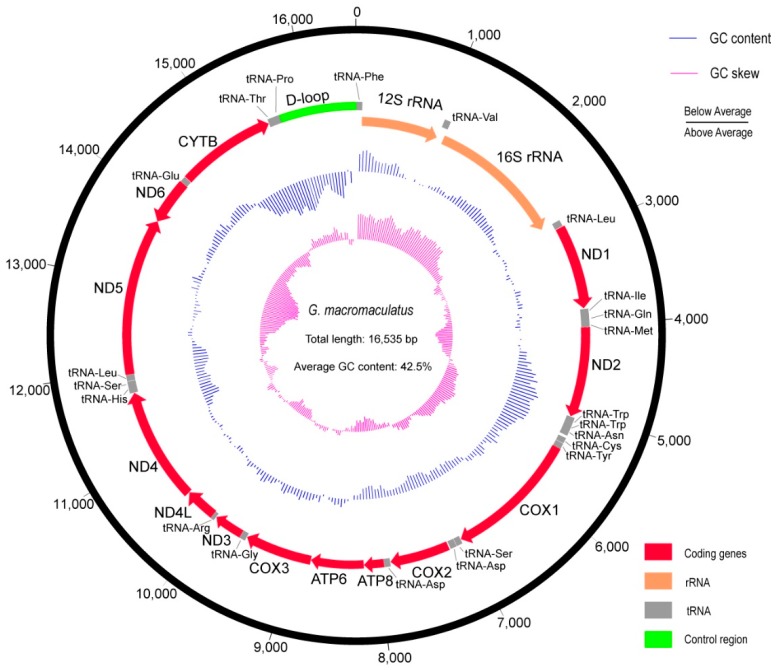
The complete mitochondrial genome of *Glyptothorax macromaculatus*. The inner blue and purple bars indicate deviations in the GC content and GC skew, respectively, in each 500-bp window across the entire mitochondrial genome.

Our incomplete mitochondrial genome assembly was ~10,000 bp, with several gaps totaling ~6000 bp. To amplify these incomplete regions, we designed 12 PCR primer pairs (Table S2). Using these primers, we obtained the complete mitochondrial genome of *G. macromaculatus* (Figure 1; GenBank accession no. MH213458).

The complete mitochondrial genome of *G. macromaculatus* was 16,535 bp, with an overall GC content of 42.5%. Our annotation identified one control region (D-loop), two ribosomal RNA (rRNA) encoding regions (12S and 16S rRNA), 22 transfer RNAs (tRNAs), and 13 protein-coding genes (*nd1*, *nd2*, *cox1*, *cox2*, *atp8*, *atp6*, *cox3*, *nd3*, *nd4l*, *nd4*, *nd5*, *nd6,* and *cytb*; Figure 1, Table 1). The structural map of the mitochondrial genome of *G. macromaculatus* indicated that most of the genes were encoded on the heavy strand (H-strand), while only a few genes were encoded on the light strand (L-strand; Figure 1, Table 1). 

The start codon of each open reading frame (ORF) encoding mitochondrial protein-coding genes in *G. macromaculatus* was typically ATG, with the exception of *cox1*, where the start codon was GTG. The stop codons of these ORFs were either TAA or Txx.

### 3.2. Phylogenetic Relationships and Divergence Times in the Sisoridae

Our multiple sequence alignment of 27 complete mitochondrial genomes, including three *Liobagrus* outgroups and 24 sisorids, comprised 17,092 aligned sites. After eliminating highly divergent regions, 15,655 sites remained and were used for phylogenetic analysis.

**Figure 2:**
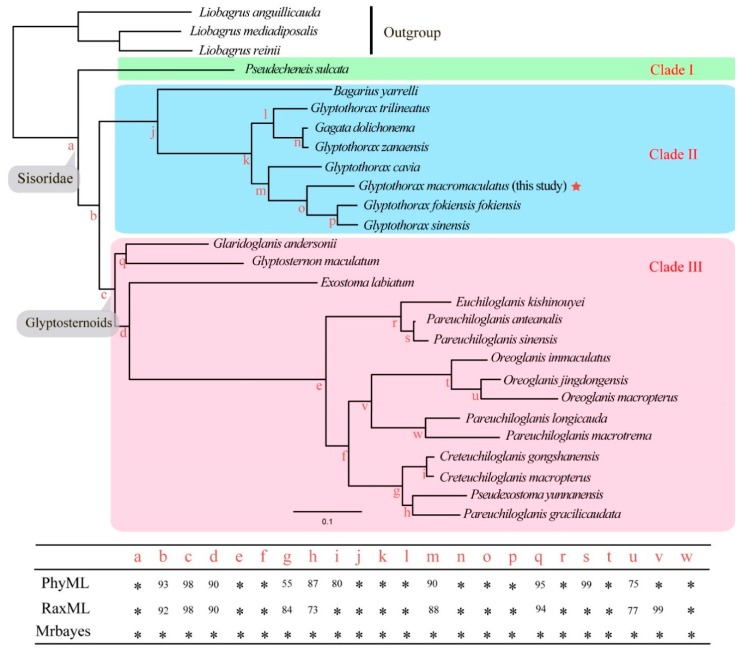
A representative molecular phylogeny of the sisorid fishes generated by maximum likelihood. *Glyptothorax macromaculatus* is indicated with a red star. The red letters (“a” to “w”) represent the support values for each node as detailed in the table; * in the table indicates 100% bootstrap support or Bayesian posterior probability. Scale bar represent nucleotide substitutions per site.

**Figure 3:**
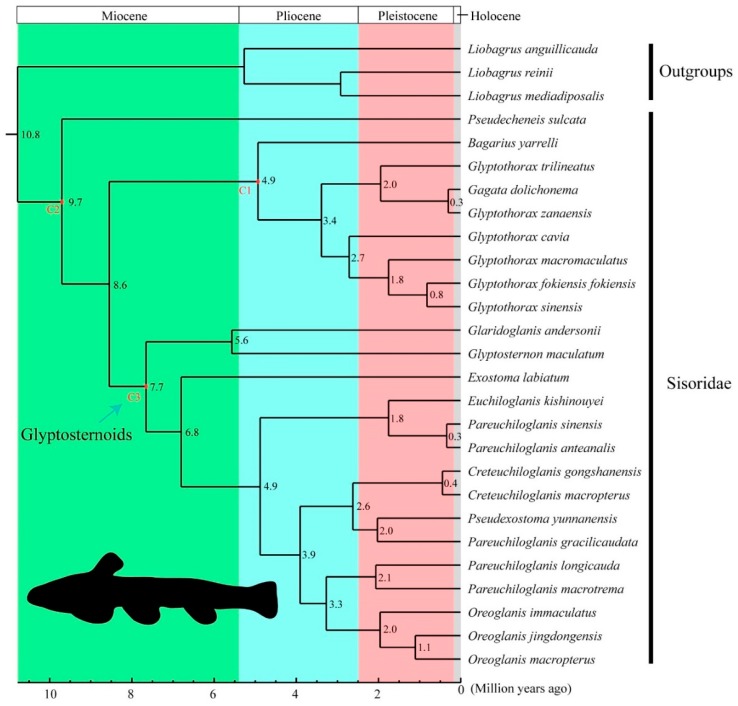
The molecular timing of species divergences within the Sisoridae, based on an analysis of complete mitochondrial genomes. C1, C2, and C3 are the three fossil-calibrated time points (see details in the text under Section 2.2).

In all phylogenies, *G. macromaculatus* was recovered in a well-supported monophyletic Sisoridae (Figure 2), a clade sister to the glyptosternoids. The genus *Glyptothorax* was well-supported but paraphyletic (Figure 2). Based on our fossil-calibrated phylogeny, most sisorid species appeared around 1~10 Ma, a period spanning the Miocene and Holocene. The divergence of *G. macromaculatus* from its MRCA was estimated to have occurred around 1.8 Ma in the Pleistocene (Figure 3).

### 3.3. The Nucleotide Substitution Rate Increased in the Glyptosternoid Fishes

**Figure 4:**
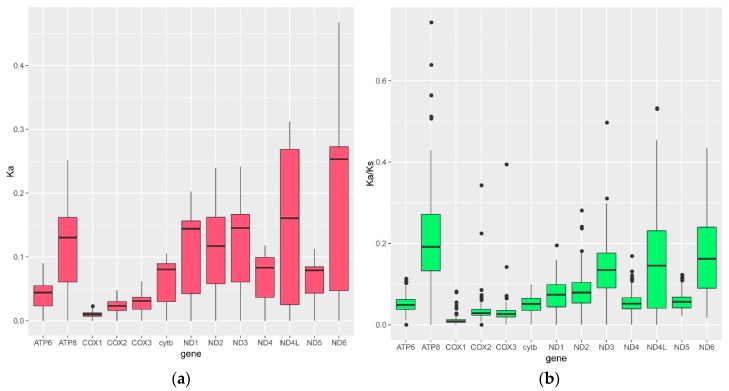
(**a**) *Ka* and (**b**) *Ka*/*Ks* for the 13 mitochondrial protein-coding genes. The average value of *Ka* is greatest for *nd6,* and the average value of *Ka/Ks* is greatest for *atp8*, suggesting that *atp8* has evolved more quickly than the other mitochondrial genes.

Of all average values of *Ka* and *Ka*/*Ks* across the 13 mitochondrial protein-coding genes, *nd6* had the largest average *Ka*, and *atp8* had the largest average *Ka*/*Ks* (Figure 4), implying that *atp8* might evolve more quickly than the other mitochondrial protein-coding genes.

We identified no significant differences between the one- and two-ratio models of ω for most mitochondrial genes (Table 2). This suggested that ω did not change more quickly than expected along the glyptosternoid branch. However, most ω values under both the models were much less than 1, suggesting that strong purifying selection drove the evolution of the mitochondrial genome in these fishes. 

Interestingly, the average *Ka*/*Ks* for *atp6* was substantially higher along the glyptosternoid branch than along any other branch (ω2 = 2.42; Table 2), implying that *atp6* may have been subjected to positive selection during the initial speciation of the glyptosternoid fishes. Both the LR and the χ^2^ tests indicated that the branch-site model of *atp6* (i.e., allowing *Ka/Ks* to vary at each site) fit the data better than the alternative hypothesis (i.e., assuming a uniform *Ka/Ks* across all sites). Our results suggested that the third codon of *atp6* had been subjected to significant positive selection along the glyptosternoid branch (*p* < 0.05).

## 4. Discussion

### 4.1. Comparison between the Complete Mitochondrial Genomes of G. macromaculatus and Other Vertebrates

The structure of the mitochondrial genome of *G. macromaculatus* was similar to those of other vertebrates, such as humans, mice, and other fishes [29,30,31,32,33,34,35]: 13 protein-coding genes, two rRNA genes, 22 tRNA genes, and one control region. The length and arrangement of the mitochondrial protein-coding genes in *G. macromaculatus* (Figure 1) was similar to those of other vertebrates, particularly other fishes [36,37], suggesting that the structure of the mitochondrial genome might be conserved across vertebrates. 

In many bony fishes, ATG is the start codon of all mitochondrial protein-coding genes except *cox1* (start codon: GTG) [31,32,35,36,37]. This pattern was also observed in *G. macromaculatus*. The uniform change to GTG in *cox1* across many fish species may indicate the early origin of this gene.

Although similar sequence patterns were identified in *G. macromaculatus* and other fishes [29,30,31,32,33,34,35], slight differences were also observed. For example, the stop codon Txx has only been observed in *Liobagrus obesus* [37]. However, only the stop codons TAA and Txx were identified in *G. macromaculatus*; the stop codon TAx was absent.

### 4.2. Molecular Phylogeny of the Sisorid Catfishes

Some previous phylogenetic studies included only a few Chinese sisorid catfishes, and thus generated only limited phylogenies of these fishes [2,3,4,5,8]. Other studies included more species, but their analyses were based on few mitochondrial genes, resulting in poorly resolved estimates of the phylogenetic relationships within the Sisoridae [2,3,4,5,6,7,8]. Our study, based on the whole mitochondrial genomes of the 12 Chinese genera in the Sisoridae, recovered a robust and well-supported phylogenetic topology.

In our phylogeny, the monophyly of the Sisoridae was strongly supported, which is consistent with previous studies [2,3,4,5,6,7,8]. The Sisoridae clustered into two major lineages in Guo et al., (2004, 2005) [3,4]: one lineage included the genera *Gagata*, *Bagarius,* and *Glyptothorax*, while the other included the genus *Pseudecheneis* and the glyptosternoids. However, this arrangement was rejected by our results. Our analysis recovered *Pseudecheneis* basal to the remainder of the Sisoridae (Clade I in Figure 2), consistent with some previous studies [5,8,9,10]. The remaining fishes fell into two major clades, one clade including the genera *Gagata*, *Bagarius,* and *Glyptothorax* (Clade II in Figure 2), and the other including the glyptosternoids (genera *Glaridoglanis*, *Glyptosternon*, *Exostoma*, *Euchiloglanis*, *Pareuchiloglanis*, *Oreoglanis*, *Creteuchiloglanis*, and *Pseudexostoma;* Clade III in Figure 2). Interestingly, *Glyptothorax* was paraphyletic, with *Gagata dolichonema* well-supported in a cluster with the *Glyptothorax* species, sister to *Glyptothorax zanaensis*. This suggested that *Gagata dolichonema* may require reassignment to *Glyptothorax*. The genus *Pareuchiloglanis*, considered paraphyletic in previous studies [5,9,10], was polyphyletic here (Clade III in Figure 2), and therefore requires taxonomic revision.

Based on the novel genome obtained in this study, *G. macromaculatus* falls in the *Glyptothorax* with good support (Clade III in Figure 2). This is the first report of the phylogenetic position of this species within the Sisoridae.

### 4.3. Molecular Dating

The divergence time of the glyptosternoids, based on molecular evidence, has been subjected to debate [4,8,9,10]. For example, Guo et al., (2005) estimated the origin of the glyptosternoids at the Oligocene-Miocene boundary (~19–24 Ma), based on *cytb* sequences [4], but Ma et al., (2015) predicted a much later appearance (~5.5 Ma) [10]. Additional estimates of this divergence time include 24 Ma [4], 5.5 Ma [8], 7.0 Ma and ~9.8 Ma [9]. Our estimate, ~7.7 Ma (Figure 3), is consistent with the most recent of these reports.

The Chinese sisorid fishes are endemic to high-altitude rivers of the Tibetan Plateau [2,8,9,10]. The uplift of the Tibetan Plateau in the Miocene generated complex geographic isolations and connections, and has been hypothesized to have had a crucial impact on sisorid speciation [8]. Our estimated date of sisorid divergence supports this hypothesis, as this date coincided with the uplift of the Tibetan Plateau. Beginning about 20 Ma, a rapid uplift began in southern Tibet, and the plateau had almost reached its present elevation by 8 Ma [38]. The uplifting of northern edge has been estimated to have occurred later, between 13.7 and 9 Ma [39]. These important tectonic events substantially changed local landmasses and river distributions [40], which in turn could have accelerated the speciation of river-dwelling sisorid catfishes.

### 4.4. Adaptations to the High Elevation

The most outstanding feature of the Tibetan Plateau is its high elevation, an average altitude of more than 4000 m. As well as high elevation, the Tibetan Plateau has a low average temperature and is typically hypoxic. Mitochondrial protein-coding genes play a crucial role in ATP generation, supporting many energy-consuming processes, such as muscle contraction and ion pumping [41,42]. It is therefore possible that exposure to hypoxia and low temperatures over evolutionary time may have affected the rates of nucleotide substitution in the mitochondrial protein-coding genes of the glyptosternoid fishes. Indeed, signals of positive selection have been observed in the mitochondrial genomes of goats [43], Tibetan antelope [44], Tibetan asses [45], Tibetan horses [46], pikas [47], Chinese snub-nosed monkeys [48], and schizothoracine fishes (Cyprinidae) [49].

Here, the average *Ka*/*Ks* varied across the mitochondrial genes, with the *Ka/Ks* of *atp8* being the most variable. This was consistent with the previously identified *Ka/Ks* ratios of two cyprinine species (*Sinocyclocheilus grahami* and *S. altishoulderus*), also restricted to the Tibetan Plateau [36]. Our results indicated that *atp6* may have been subjected to positive selection along the branch ancestral to the glyptosternoids. However, our results were incongruent with a previous study, which showed that *cox1* had been subjected to positive selection in the glyptosternoid fishes [10] (Table 2). 

In summary, although our results suggested that most mitochondrial protein-coding genes had not been subjected to positive selection, *atp6* might have changed rapidly to adapt to the high elevation of the Tibetan plateau. However, as effective population size [10] and life history [11,12] may also affect *Ka/Ks* values, more data, including population genetics and life history, are required to substantiate our present results.

## 5. Conclusions

Here, we assembled the first complete mitochondrial genome of *G. macromaculatus* and used this novel genome to determine the phylogenetic position of this species within the sisorid catfishes. Our phylogeny, which included representatives of all the 12 genera of Chinese sisorid catfishes, was robust and well-supported. Based on our preferred topology, we resolved some controversial issues and clarified some genus-level relationships within the Sisoridae. Interestingly, our molecular dating analysis indicated that the uplift of the Tibetan Plateau may have accelerated the speciation of the Chinese sisorid catfishes. Specifically, this uplift may have increased the nucleotide substitution rate of certain mitochondrial protein-coding genes (e.g., *atp6*), promoting the adaption of the sisorid fishes to the high elevations and low temperatures of the Tibetan Plateau. 

## Figures and Tables

**Table 1:** Characteristics of the mitochondrial genome of *Glyptothorax macromaculatus*.

				Codon			
Gene/Element	from	to	Length (bp)	Start	Stop	Intergenic Nucleotides	Strand
tRNA-Phe	1	69	69				H
12S rRNA	70	1021	952			5	H
tRNA-Val	1027	1098	72				H
16S rRNA	1099	2774	1677				H
tRNA-Leu (UUR)	2775	2849	76				H
*nd1*	2850	3824	975	ATG	TAA	5	H
tRNA-Ile	3830	3901	72			−1	H
tRNA-Gln	3901	3971	71			−2	L
tRNA-Met	3970	4038	69			2	H
*nd2*	4041	5085	1045	ATG	T--		H
tRNA-Trp	5086	5156	71			2	H
tRNA-Ala	5159	5227	69			1	L
tRNA-Asn	5229	5301	73				
tRNA-Cys	5334	5396	63			6	L
tRNA-Tyr	5403	5471	69			1	L
*cox1*	5473	7023	1551	GTG	TAA	1	H
tRNA-Ser (UCN)	7025	7093	69			5	L
tRNA-Asp	7099	7170	72			15	H
*cox2*	7186	7876	691	ATG	T--		H
tRNA-Lys	7877	7950	74			1	H
*atp8*	7952	8119	168	ATG	TAA	−8	H
*atp6*	8110	8793	684	ATG	TAA	−1	H
*cox3*	8793	9576	784	ATG	T--		H
tRNA-Gly	9577	9648	72				H
*nd3*	9649	9997	349	ATG	T--		H
tRNA-Arg	9998	10,067	70				H
*nd4l*	10,068	10,359	292	ATG	T--	−2	H
*nd4*	10,358	11,738	1381	ATG	T--		H
tRNA-His	11,739	11,808	70				H
tRNA-Ser (AGY)	11,809	11,876	68			5	H
tRNA-Leu (CUN)	11,882	11,954	73				H
*nd5*	11,955	13,777	1823	ATG	TAA		H
*nd6*	13,778	14,296	519	ATG	TAA		L
tRNA-Glu	14,297	14,365	69			5	L
*cytb*	14,371	15,508	1138	ATG	T--		H
tRNA-Thr	15,509	15,576	68			2	H
tRNA-Pro	15,579	15,648	70				L
D-loop	15,649	16,535	887				-

**Table 2:** Models of selection pressure on the mitochondrial protein-coding genes of the glyptosternoid fishes.

Gene	ω0	ω1	ω2	*p* Value
*atp6*	0.03793	0.03687	2.4219	0.009 *
*atp8*	0.13819	0.13814	0.13939	0.13
*cox1*	0.01162	0.01182	0.0001	0.15
*cox2*	0.02230	0.02230	0.0001	0.14
*cox3*	0.02792	0.02825	0.0001	0.18
*cytb*	0.03201	0.03230	0.01563	0.44
*nd1*	0.05152	0.05250	0.00927	0.08
*nd2*	0.6942	0.07010	0.03519	0.36
*nd3*	0.6969	0.6920	+∞	0.27
*nd4*	0.4179	0.04198	0.03187	0.73
*nd4l*	0.07551	0.07824	0.0001	0.11
*nd5*	0.05333	0.5386	0.1308	0.20
*nd6*	0.07424	0.07390	0.08247	0.88

Note: The ratio of non-synonymous to synonymous substitutions (*Ka*/*Ks*) was calculated using codon alignments of the glyptosternoid fishes with other sisorid species. ω0 is the average *Ka*/*Ks* ratio across all branches (one-ratio model); ω1 is the average *Ka*/*Ks* ratio along the non-glyptosternoid branches and ω2 is the *Ka*/*Ks* ratio along the glyptosternoid branches (two-ratio model). * indicates a significant difference between the two-ratio and one-ratio models (*p* < 0.05).

## Supplementary Materials

The following materials are available online at http://www.mdpi.com/2073-4425/9/6/282/s1. Table S1: The downloaded complete mitochondrial genomes from NCBI; Table S2: Sequences of the designed 12 primer pairs for amplification of the *G. macromaculatus* mitochondrial genome.

## Abbreviations

*atp6*	Adenosine triphosphate synthase subunit 6
*atp8*	Adenosine triphosphate synthase subunit 8
bp	Base pair
*cox1*	Mitochondrial encoded cytochrome c oxidase 1
*cox2*	Mitochondrial encoded cytochrome c oxidase 2
*cox3*	Mitochondrial encoded cytochrome c oxidase 3
*cytb*	Cytochrme b
*Ka*	Nonsynonymous substitutions
kb	kilobase
*Ks*	Synonymous substitutions
*nd1*	NADH-Ubiquinone Oxidoreductase Chain 1
*nd2*	NADH-Ubiquinone Oxidoreductase Chain 2
*nd3*	NADH-Ubiquinone Oxidoreductase Chain 3
*nd4*	NADH-Ubiquinone Oxidoreductase Chain 4
*nd4l*	NADH-Ubiquinone Oxidoreductase Chain 4L
*nd5*	NADH-Ubiquinone Oxidoreductase Chain 5
ORF	Open reading frame
PCR	Polymerase chain reaction
