# Supplementary material for: The Complete Mitochondrial Genome of Glyptothorax macromaculatus Provides a Well-Resolved Molecular Phylogeny of the Chinese Sisorid Catfishes
Source: Genes (Basel). 2018 Jun 4;9(6):282. doi: 10.3390/genes9060282 (PMC6027347; doi:10.3390/genes9060282)
Supplement: Supplementary file 1 [file genes-09-00282-s001.zip › Table S1 revised.docx]

**Table S1** The downloaded complete mitochondrial genomes from NCBI.

| **Species** | **Accession number** | **For the phylogeny analysis** |
| --- | --- | --- |
| *Liobagrus anguillicauda* | JQ026256 | Outgroup |
| *Liobagrus mediadiposalis* | KR075136 | Outgroup |
| *Liobagrus reinii* | AP012015 | Outgroup |
| *Pseudecheneis sulcata* | JQ026259 | Ingroup |
| *Bagarius yarrelli* | JQ026260 | Ingroup |
| *Glyptothorax trilineatus* | JQ026262 | Ingroup |
| *Gagata dolichonema* | JQ026250 | Ingroup |
| *Glyptothorax zanaensis* | NC_029709 | Ingroup |
| *Glyptothorax cavia* | NC_034921 | Ingroup |
| *Glyptothorax macromaculatus* | This study | Ingroup |
| *Glyptothorax fokiensis fokiensis* | JQ917224 | Ingroup |
| *Glyptothorax sinensis* | NC_024672 | Ingroup |
| *Glaridoglanis andersonii* | JQ026254 | Ingroup |
| *Glyptosternon maculatum* | JQ026251 | Ingroup |
| *Exostoma labiatum* | JQ026255 | Ingroup |
| *Euchiloglanis kishinouyei* | NC_021598 | Ingroup |
| *Pareuchiloglanis anteanalis* | KP872692 | Ingroup |
| *Pareuchiloglanis sinensis* | KP872695 | Ingroup |
| *Oreoglanis immaculatus* | KP872690 | Ingroup |
| *Oreoglanis jingdongensis* | KP872691 | Ingroup |
| *Oreoglanis macropterus* | JQ026261 | Ingroup |
| *Pareuchiloglanis longicauda* | KP872693 | Ingroup |
| *Pareuchiloglanis macrotrema* | KP872694 | Ingroup |
| *Creteuchiloglanis gongshanensis* | KP872697 | Ingroup |
| *Creteuchiloglanis macropterus* | KP872682 | Ingroup |
| *Pseudexostoma yunnanensis* | JQ026258 | Ingroup |
| *Pareuchiloglanis gracilicaudata* | JQ026257 | Ingroup |
