# Supplementary material for: The Complete Mitochondrial Genome of Glyptothorax macromaculatus Provides a Well-Resolved Molecular Phylogeny of the Chinese Sisorid Catfishes
Source: Genes (Basel). 2018 Jun 4;9(6):282. doi: 10.3390/genes9060282 (PMC6027347; doi:10.3390/genes9060282)
Supplement: Supplementary file 1 [file genes-09-00282-s001.zip › Table S2 revised.docx]

**Table S2**. Sequences of the designed 12 primer pairs for amplification of the *G. macromaculatus* mitochondrial genome.

| **Locus** | **Forward (F) Primer (5’-3’)** | **Reverse (R)Primer (5’-3’)** |
| --- | --- | --- |
| MT1 | CCCGGATTTGGTATAATCTCCC | TCCTTTAGAAGCGGGTTGAATGA |
| MT2 | AACACCTTTTCTGGTTCTTCGG | GGAGTTGCACCAAGAGTTTTTG |
| MT3 | AAACACCACATTCTTTGACCCAG | GGTGAGTAGGAGGGAGGTGG |
| MT4 | AATTTGGGTTACATCCATGATTGC | TATCAACTGATGATGATTTCTTACTG |
| MT5 | AACCCTCCCGCTAATAGGAATT | ACCCCCACAGTTTGTGTCCTT |
| MT6 | AACTCATGAGAACTACCCCAAAT | TTATGGCCCTGAAATAGGAACC |
| MT7 | TGAAGTCAACGACCCCCATTTAAC | TGTTGGCTGATTTCGTAGGCCAAT |
| MT8 | CGTAAAAGCCATAGGCCATCAA | GGGGATTAATGGTGTGGGAGTTCC |
| MT9 | ATGATACTGAAGCTACGAATATAC | GGCTAAAGGTCGAATAAATAGGC |
| MT10 | ACCATTACACTACTACCAATAATA | ATGATGACATGTAGTCCGTGGAAG |
| MT11 | ACACTGCTAACCTAACAGCAGGCC | TAGGAGGCAGACGGCGAGGAAGG |
| MT12 | TGAAACAATTAGCCTATTTATTCG | TTCAAATCCGAAATGGTGTTCTG |
